# Blood free Radicals Concentration Determined by Electron Paramagnetic Resonance Spectroscopy and Delayed Cerebral Ischemia Occurrence in Patients with Aneurysmal Subarachnoid Hemorrhage

**DOI:** 10.1007/s12013-017-0820-7

**Published:** 2017-09-25

**Authors:** Grzywna Ewelina, Stachura Krzysztof, Moskala Marek, Kruczala Krzysztof

**Affiliations:** 1 Department of Neurosurgery and Neurotraumatology, Jagiellonian University Medical College, Krakow, Poland; 2 Faculty of Chemistry, Jagiellonian University, Krakow, Poland

**Keywords:** Aneurysmal subarachnoid hemorrhage, Blood free radicals, Cerebral vasospasm, Delayed cerebral ischemia, Electron paramagnetic resonance, EPR

## Abstract

Pathophysiology of delayed cerebral ischemia and cerebral vasospasm following aneurysmal subarachnoid hemorrhage is still poorly recognized, however free radicals are postulated as one of the crucial players. This study was designed to scrutinize whether the concentration of free radicals in the peripheral venous blood is related to the occurrence of delayed cerebral ischemia associated with cerebral vasospasm. Twenty-four aneurysmal subarachnoid hemorrhage patients and seven patients with unruptured intracranial aneurysm (control group) have been studied. Free radicals in patients’ blood have been detected by the electron paramagnetic resonance (CMH.HCl spin probe, 150 K, ELEXSYS E500 spectrometer) on admission and at least 72 h from disease onset. Delayed cerebral ischemia monitoring was performed by daily neurological follow-up and transcranial color coded Doppler. Delayed cerebral ischemia observed in six aneurysmal subarachnoid hemorrhage patients was accompanied by cerebral vasospasm in all six cases. No statistically significant difference in average free radicals concentration between controls and study subgroups was noticed on admission (*p* = .3; Kruskal–Wallis test). After 72 h free radicals concentration in delayed cerebral ischemia patients (3.19 ± 1.52 mmol/l) differed significantly from the concentration in aneurysmal subarachnoid hemorrhage patients without delayed cerebral ischemia (0.65 ± 0.37 mmol/l) (*p* = .012; Mann–Whitney test). These findings are consistent with our assumptions and seem to confirm the role of free radicals in delayed cerebral ischemia development. Preliminary results presented above are promising and we need perform further investigation to establish whether blood free radicals concentration may serve as the biomarker of delayed cerebral ischemia associated with cerebral vasospasm.

## Introduction

Regardless the high disability and mortality that is related with the severity of aneurysmal subarachnoid hemorrhage (aSAH) by itself, the final long-term outcome is greatly affected by the aSAH complications. Delayed cerebral ischemia (DCI) associated with cerebral vasospasm remains a major cause of disability and death in aSAH survivors [1]. Typically it develops between days 4 and 10 after aneurysm rupture and subarachnoid bleeding [1, 2]. Despite extensive basic studies the pathophysiology of DCI and cerebral vasospasm remains recognized incompletely. Generally, it is known, that the presence of blood in the subarachnoid space results in contact of hemoglobin decay products with the abluminal side of the vessels; this initiates a multifaced cascade of events culminating in arterial narrowing. Among different mechanisms involved in this process free radicals and oxidative stress are postulated as one of the crucial players [3, 4]. Based on this knowledge many attempts with free radicals scavengers (tirilazad mesylate just to mentioned most often investigated) were made to prevent and/or to treat DCI and cerebral vasospasm. In experimental studies positive effects were found, however, in human none of them are recommended as a routine therapy of proven efficacy [5, 6]. Still very little is known about the significance of markers related to oxidative stress that may indirectly prove the role of free radicals in the pathophysiology of DCI and cerebral vasospasm. Elevated free fatty acids (markers of lipids peroxidation) were noticed in cerebrospinal fluid (CSF) of patients with DCI [7]. Pyne-Geithman et al. investigated antioxidant response in ten aSAH patients and found an elevated activity of glutathione peroxidase in CSF of patients with vasospasm [8]. The general conclusion of these studies is in patients with DCI and cerebral vasospasm there is an increased oxidative stress and an imbalance between prooxidants and antioxidants. The electron paramagnetic resonance (EPR) combining with the spin trapping technique is a method of choice to investigated free radicals [9, 10]. The application of EPR to studies on human blood have enabled discovery of the mechanisms of formation and decay of globin-based free radicals [11]. To the best of our knowledge, the concentration of free radicals in blood of patients with subarachnoid hemorrhage wasn’t analyzed so far.

This study was designed to scrutinize whether the concentration of free radicals in the peripheral venous blood increases in the course of aSAH and whether it is related to the occurrence of DCI associated with cerebral vasospasm. We aimed particularly to determine if the blood free radicals concentration may serve as the biomarker of DCI associated with cerebral vasospasm in the aSAH patients. For this purpose the correlation between free radicals concentration, mean flow velocity (MFV) in the cerebral arteries and DCI occurrence was studied.

## Material and Methods

### Study Group

Twenty-four patients admitted to the Neurosurgery Department of University Hospital in Krakow (Poland) consecutively between November 2012 and August 2013 with diagnosis of aneurysmal subarachnoid hemorrhage were studied. Study group consisted of 11 women (45.83%) and 13 men (54.17%) median age of 58.5 years (range 28–80 years). The inclusion criteria were as follow: age between 18 and 80 years, diagnosis of subarachnoid hemorrhage (based on head non-contrast computed tomography or lumbar puncture), intracranial aneurysm (IA) presence confirmed in the angio-CT (computed tomography), angio-magnetic resonance imaging (MRI), or cerebral digital subtractive angiography (DSA) and disease onset less then 48 h before admission. The exclusion criteria were additionally: known medical conditions related with increased formation of free radicals (trauma, inflammatory disease, and malignancy), the history of recent (less then 4 weeks) immunosuppressive or steroid treatment, critical condition of the patient on admission (grade 5 in Hunt and Hess scale, Glasgow Coma score (GCS) of 3–5), pregnancy and breastfeeding, concomitant participation in other trials.

Patient’s medical history was collected based on the interview (if possible) and provided medical records. A general physical and neurological examination was performed by the study neurosurgeon on admission, and the patients were graded in the Hunt and Hess [12] and World Federation of Neurological Surgeons (WFNS) scale [13]. Two scales to follow the clinical condition of aSAH patients were used since Hunt and Hess grading system is widely known and well entrenched in the literature and WFNS contains the component of GCS that closely correlates with clinical outcome [14]. The initial head CT scan was analyzed in each case and the grade in the Fisher scale was determined [15]. In the course of hospitalization neurological examination was performed on everyday basis or more frequently if required by the changes of the patient’s clinical condition. Patients’ 30-day outcome was determined with use of modified Rankin Scale (mRS) [16]. All aSAH patients were administered oral nimodipine as the prevention of DCI associated with cerebral vasospasm [17].

This study was conducted in accordance with the ethical standards laid down in the Declaration of Helsinki (1964) and its design was approved by the local University Ethical Committee (protocol number KBET/152/B/2012).

### Control Group

Seven patients admitted to the Neurosurgery Department with diagnosed unruptured IA (angio-CT/MRI or DSA) were recruited as the control subjects. Four women (57.14%) and three men (42.86%) median age of 55 years (range 32–65 years) were included. The same exclusion criteria as in the study group were applied. Patient’s medical history was collected and general physical and neurological examination was performed by the study neurosurgeon on admission.

### Blood Samples and EPR Spectroscopy

Peripheral venous blood was collected into the 2.5 ml l-heparin vacutainer containing 1.25 ml of stock solution. Blood sampling was carried out twice in the study group—on days: 0 (admission), 3–7 and once in the control group (admission).

To overcome the technical difficulties associated with low concentration and short life time of free radicals in biological samples, the solution of spin probe CM-H·HCl (1-hydroxy-3-methoxycarbonyl-2,2,5,5-tetramethylpyrrolidine·HCl, C_10_H_19_NO_3_·HCl) and Ar-flushed 20 mM Krebs-HEPES pH.7.4 buffer was used (stock solution). Prepared blood samples were injected into the quartz high precision EPR tubes and immediately frozen in liquid nitrogen (LN). At the same time reference samples containing stock solution were prepared.

**Fig. 1:**
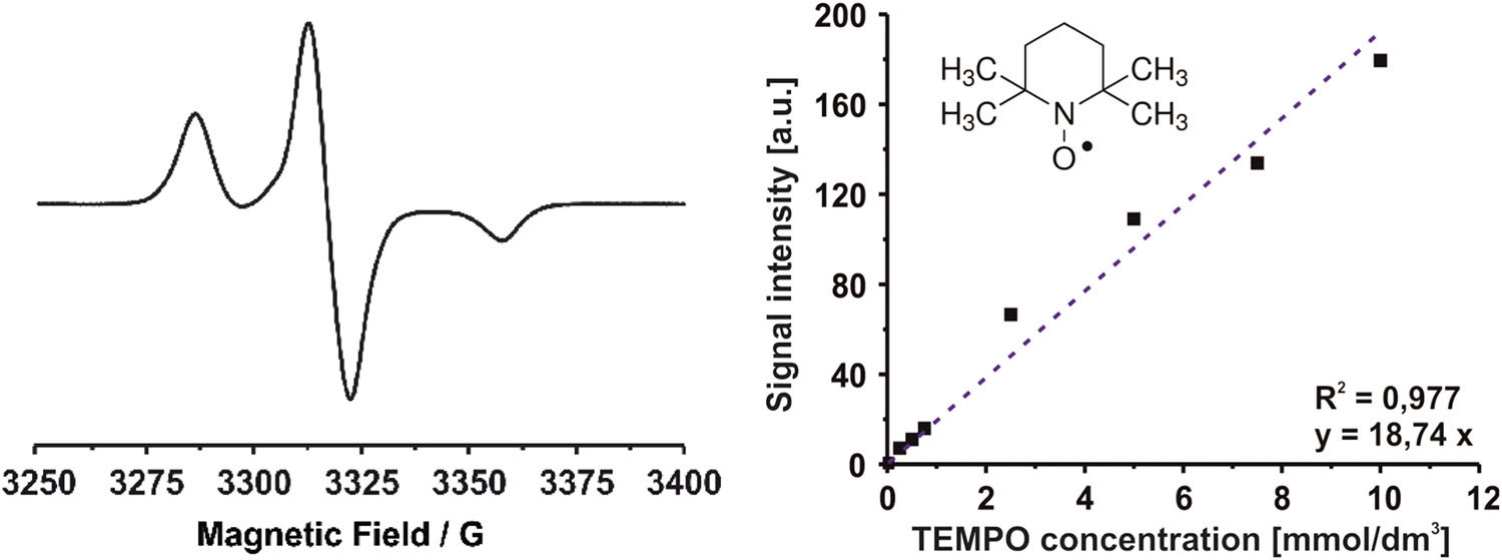
X-band EPR spectrum of the oxidized CMH spine probe (CM^•^) registered at 150 K (*left*) and calibration curve used for concentration determination (*right*). In the onset the formula of TEMPO, correlation parameter *R* ^2^ and question of straight line are given in the onset

All EPR measurements were performed at 150 K temperature (ER 4111 VT variable temperature unit) by using the Bruker Elexsys E-500 X-band spectrometer equipped with ER TM_110_ cavity, operating at 9.4 GHz, 2 mW power and 0.1 mT modulation amplitude. Free radicals concentration was determined by comparison of the integral signal intensity of the investigated samples with that of the TEMPO ([2,2,6,6-tetramethylpiperidin-1-yl]oxyl) standard solutions of know concentrations of the paramagnetic centers (Fig. 1).

### DCI and Cerebral Vasospasm Monitoring

DCI was diagnosed according to recognized clinical criteria [1, 18].

All aSAH patients were monitored for the presence of cerebral vasospasm with use of transcranial color-coded Doppler (TCCD). Study design assumed TCCD examination in all aSAH patients every other day, starting from the admission day or more frequently if required by the changes of the patient’s clinical condition. Control subjects were examined with use of TCCD once—on admission. TCCD was performed by the trained neurosurgeon (certified skills) with use of Aloka ProSound 3500SX ultrasound. Cerebral vasospasm was diagnosed according to published criteria [19, 20]. This way to monitor patients was chosen based on recommendations of American Heart Association/American Stroke Association published in Stroke in 2012 [18], with the awareness that TCCD has a lower sensitivity and specificity to diagnose arterial narrowing then CT/MRI angiography or digital subtraction angiography [1].

### Statistical Analysis

Quantitative results are presented as median with lower and upper quartile and minimum and maximum value. Data were compared across groups using Mann–Whitney test (two groups) or the Kruskal–Wallis test with the Bonferroni post-hoc tests (more than two groups) due to the lack of normality or the ordinal scale of responses. The Shapiro–Wilk test was used to assess normality.

*p* values <.05 were considered statistically significant. The analyzes were performed using SPSS Statistics v.21 (IBM, New York, USA).

## Results

### Characteristics of the aSAH Patients

The clinical profile of studied aSAH patients is presented in Table 1. There were four deaths in the study group (16.67%). One patient died due to severe aSAH (ruptured BA aneurysm) and massive edema in the posterior fossa. Another patient developed pneumonia and died despite aggressive treatment. Two patients developed DCI associated with severe cerebral vasospasm and, consequently, cerebral infarction resulting in massive brain edema.

**Table 1:** Baseline characteristics of the study group

Patients with aneurysmal SAH	*n* = 24
Age median (year)	58.5
Sex	
Female	11 (45.83%)
Male	13 (54.17%)
Comorbidities	
Arterial hypertension	15 (62.5%)
Coronary artery disease	4 (16.67%)
Peripheral artery disease	4 (16.67%)
History of stroke	3 (12.5%)
Diabetes mellitus type 2	4 (16.67%)
History of smoking	11 (45.83%)
History of alcohol abuse	3 (12.5%)
On admission	
Median of Hunt and Hess grade	3
Median of WFNS grade	2
Median of Fisher grade	3
Coexisting intracerebral hematoma needed surgical evacuation (%)	4 (16.67%)
Aneurysm distribution (no.)—ruptured only	
Anterior communicating artery (AcomA)	10 (41.67%)
Middle cerebral artery (MCA)	5 (20.83%)
Internal carotid artery (ICA)	4 (16.67%)
Basilar artery (BA)	3 (12.5%)
Other	2 (8.33%)
Received aneurysm treatment	
Coiling	11 (45.83%)
Clipping	13 (54.17%)
Other	0
None	0
White blood cells (x10^3^/μl)	6.75 ± 1.85
Average mRS 30 days post aSAH ± SD	3.1 ± 1.8
Deaths	4 (16.67%)

### Characteristics of the DCI Patients

Six aSAH patients (25% of study group) developed DCI 5–10 days after subarachnoid bleeding. DCI group consisted of three men and three women, median age of 56.5 years (range 28–73 years). None of DCI patients suffered from coronary artery disease, peripheral artery disease, or diabetes mellitus. Sixty-seven percent of DCI patients has diagnosed preexisting arterial hypertension. Three DCI patients were active cigarette smokers (67%) and one patient had a history of alcohol abuse (17%). The median of Hunt and Hess grade on admission in DCI patients was 3 and the median of WFNS grade was 1.5. The median of Fisher grade for the initial head CT scan was 3. In three DCI patients (50%) the cause of subarachnoid hemorrhage was the rupture of the anterior communicating artery (ACoA) aneurysm, in another two (33%)—the rupture of middle cerebral artery (MCA) aneurysm and in one (17%)—internal carotid artery (ICA) aneurysm rupture. Four DCI patients (67%) had their aneurysm secured by clipping and another two (33%)—by coiling (ACoA and ICA aneurysm). Two DCI patients (33%) died due to cerebral infarction and brain edema resulting from DCI associated with severe cerebral vasospasm. At day 30 post aSAH average mRS for DCI patients was 4.5 ± 1.2. This outcome have been worse than the other patients’ in the study group and the difference is statistically significant (*p* = .018, Mann–Whitney test).

### DCI and Cerebral Vasospasm

Neurological deterioration diagnosed as DCI in six aSAH patients was scored for 5, 6, 7, 14, 16, and 24 points in NIH Stroke Scale. Neurological symptoms were accompanied by the increase of MFV in anterior cerebral artery and/or middle cerebral artery meeting the TCCD criteria of cerebral vasospasm in all six cases. In two patients aggressive management of DCI resulted in resolution of neurological symptoms within 3–5 days from the onset of symptoms. No endovascular interventions were implemented. Simultaneously decrease of MFV in serial TCCD studies was observed in these two patients that proves the resolution of cerebral vasospasm. In the remaining four patients intensive treatment of DCI did not bring improvement of their neurological status. In these patients head CT scan performed 24–48 h from the new neurological symptoms onset revealed cerebral infarction corresponding to the vessel(s) affected by cerebral vasospasm. After 6 weeks this finding was confirmed in control head CT in the DCI patients who survived (two patients).

### Blood Free Radicals

**Scheme 1:**
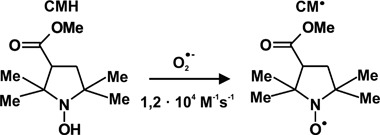
Reaction scheme of CMH spin probe with superoxide radicals

The study of the free radical in the peripheral venous blood was performed by EPR spectroscopy with use of CMH, an effective cell permeable membrane spin probe for the quantification of reactive oxygen species (ROS) [21–25]. This spin probe CMH react mostly with superoxide radicals (Scheme 1) but also peroxyl radicals, peroxynitrite, nitrogen dioxide were as the one is not sensitive to nitric oxide and hydrogen peroxide [21–25]. The oxidation of CMH leads to the formation of the paramagnetic, EPR active, 3-methoxycarbonyl-proxyl nitroxide (CM^•^) [26]. The concentration of CM^•^ is proportional to the concentration of the oxidant species, manly (both extra-cellular and intracellular) [21–25]. CMH detects both extra-cellular and intracellular superoxide radicals The typical EPR spectrum of the oxidized spin probe registered at 150 K (left) and calibration curve used for concentration determination (right) are given in Fig. 1.

Blood free radicals concentration in the aSAH patients groups (with and without DCI) and in the control group was compared. No statistically significant difference in average free radicals concentration between study groups and controls was noticed on admission (*p* = .3; Kruskal–Wallis test). After 72 h or more from the SAH onset (time window for DCI associated with vasospasm) blood free radicals concentration in DCI patients (3.18 ± 1.52 mmol/dm^3^) differed significantly from the concentration in blood of aSAH patients who didn’t developed DCI (0.65 ± 0.37 mmol/dm^3^) (*p* = .012; Mann–Whitney test) as presented in Table 2.

**Table 2:** Venous blood free radicals concentration on admission and after at least 72 h from the SAH onset

Blood free radicals concentration (mmol/dm^3^)	Study group—DCI patients	Study group—non-DCI patients	Control group	*p*
On admission				
Mean ± SEM	1.18 ± 0.48	0.55 ± 0.16	0.48 ± 0.12	.3*
95% CI	−0.13–2.50	0.20–0.90	0.19–0.78	
Median	0.48	0.34	0.49	
IQR	1.96	0.35	0.57	
After ≥72 H				
Mean ± SEM	3.18 ± 1.52	0.65 ± 0.37		.012**
95% CI	0.70–7.07	−0.19–1.49		
Median	2.07	0.34		
IQR	5.07	0.26		

*SEM* standard error of the mean, *95% CI* 95% confidence interval, *IQR* interquartile range

^*^ *p* for Kruskal–Wallis’ statistics

^**^ *p* for Mann–Whitney’s statistics

### Blood free Radicals Concentration and MFV

**Fig. 2:**
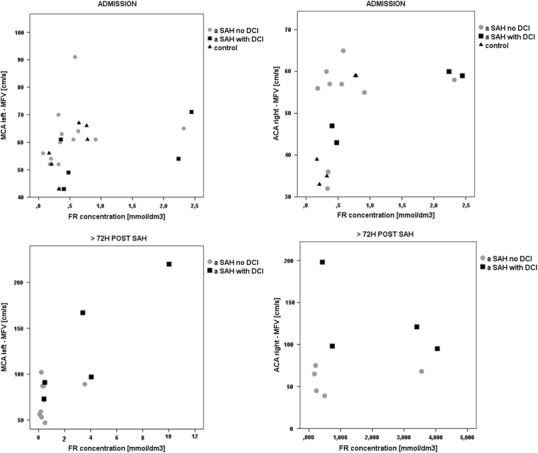
Blood free radicals concentration and mean flow velocity (MFV) in cerebral vessels of aSAH patients

Due to low number of patients in analyzed statistical groups the correlation between blood free radicals concentration and MFV in cerebral vessels of aSAH patients could not be determined. However, the graphical illustration these dependencies allows to see relation, which may be promising in the context of further research (Fig. 2).

Greater number of patients in analyzed subgroups would allow then to apply statistical tests that would express this relation in numbers.

## Discussion

DCI associated with cerebral vasospasm remains the most important single complication leading to disability and death in aSAH survivors. Signs of DCI may be reversible but may also persist or progress in situation of cerebral infarction, leading to disability or death. The most recent data reveals that DCI may develop independently of the radiological vasospasm presence. This most likely results from the impairments in the arteriolar circulation not visible angiographically, from the damage to the cerebral tissue in the first 72 h after aneurysm rupture (“early brain damage”), cortical spreading depression and microthrombosis [27–29]. Aneurysmal bleeding into the subarachnoid space initiates multifaced cascade of events resulting in inflammation and oxidative stress. Among different mechanisms involved in DCI and cerebral vasospasm development, free radicals and oxidative stress are considered to be substantial [3, 4, 30]. The excessive formation of free radicals in subarachnoid hemorrhage occurs through different pathways like extracellular hemoglobin conversions, disruption of mitochondrial respiration, and upregulation of enzymes producing free radicals: inducible nitric oxide synthase, NADPH oxidase (NOX), xanthine oxidase and other [30].

Six out of twenty-four patients in the study group developed delayed neurological deterioration accompanied by the impairments in the cerebral circulation diagnosed as delayed cerebral ischemia associated with cerebral vasospasm. This proportion is consistent with population-based studies [31].

The median of age in the study group and in the subgroup of DCI patients haven’t differed significantly (58.5 vs. 56.5 years). Some previous studies found the younger aSAH patients (<35 years of age, <50 years of age in other study) more prone to develop DCI associated with cerebral vasospasm [32, 33] while the other studies have shown higher incidence of discussed aSAH complication with the increasing age (>60 years of age) [34]. In the most recent studies patient’s age wasn’t found to be predictive for DCI associated with cerebral vasospasm [35] and it is the same in the presented material. Anyway the there is disagreement in the literature regarding the relationship between age and the risk of DCI associated with cerebral vasospasm, and it has been related to the variety of different definitions and terms used to describe this clinical phenomenon and different measurement modalities used in the studies. Similar percentage of men (23%) and women (27%) in the study group developed the DCI associated with cerebral vasospasm. Among the comorbidities included in the clinical characteristics of the study group, the history of preexisting arterial hypertension is considered to be the risk factor of DCI associated with cerebral vasospasm [36] and in our study it was more often in the DCI patients (67%) than in the entire study group (63%) but the described difference haven’t met the criteria for statistical significance. Several papers (including prospective studies) suggest cigarette smoking as the independent predictor of DCI associated with cerebral vasospasm in the aSAH patients [37, 38]. Presented results confirm this observation—it was 46% of active cigarette smokers in the study group comparing with 67% in the DCI subgroup. One retrospective study revealed that nicotine replacement therapy in active smokers with aSAH may reduce the risk of DCI associated with vasospasm, while another study of similar size have failed to repeat these results [39]. None of the components of cigarette smoke has not yet been identified as a single agent which will lead to the development of DCI. Smoking leads to the increased free radicals formation in blood [40] and this might the promising in the context of the results presented in this paper. The clinical severity of aSAH evaluated with use of Hunt and Hess scale and WFNS haven’t shown any significant difference between all aSAH patients and DCI subgroup. The median of Hunt and Hess grade on admission was three in both groups when the result ≥ 3 is considered increasing the risk of DCI associated with cerebral vasospasm [36, 41]. WFNS haven’t been proved to be predictive for DCI/cerebral vasospasm [33]. In the 1970s the relationship between the amount and distribution of blood in the subarachnoid space on the initial head CT scan and development of visible angiographically vasospasm was reported. Since then Fisher grading scale for the classification of the admission head CT scans was introduced into clinical practice and high correlation between DCI associated with cerebral vasospasm and Fisher grade 3 have been revealed. Fisher grade of 3 (the median in the study group and in the DCI subgroup) increases the odds of DCI development [15, 42–44]. Clipping was applied in more aSAH patients than coiling (13 vs. 11) in the study group [45]. More DCI patients received clipping [4] than coiling [2] of their ruptured IA. It is known that DCI develops more often after clipping then after coiling (International Subarachnoid Aneurysm Trial) and it is consistent with our observations [43]. The most recent data demonstrate it doesn’t affect the final outcome, except the aSAH patients who were treated within 4 days after the initial bleeding—in this particular group DCI is associated with poorer outcome after coiling then after clipping [46]. All aSAH patients studied in our material were treated within 48 h from the aSAH onset.

EPR technique, coupled to a specific spin probe (CMH: 1-hydroxy-3-methoxycarbonyl-2,2,5,5-tetramethylpyrrolidine) is here presented as the method of choice to gain a direct measurement of ROS in biological fluids and tissues. The correlation between EPR results and data obtained by various enzymatic assays (e.g., protein carbonyls and thiobarbituric acid reactive substances) was determined too [47]. CM-H.HCl has been previously used in studies on Alzheimer’s disease (liquid samples) and atherosclerosis (tissue samples) [26, 47–49]. Mechanisms of globin-based free radicals formation and decay in the human blood were studied and their baseline concentration was estimated at 1μmol/dm^3^ in healthy volunteers [11]. To the best of our knowledge, reports on the concentration of free radicals in the blood of patients with aneurysmal subarachnoid hemorrhage are not available. One of our aims was to investigate whether the concentration of free radicals in the blood increases in the course of aSAH and whether it is related to the occurrence of DCT/cerebral vasospasm. EPR supported by the spin probe (CMH.HCl) was used to address the issue of detection and measurements of free radicals in the human venous blood. No statistically significant difference in average free radicals concentration between controls and study groups (patients with/without DCI, less than 48 h of SAH onset) was demonstrated on admission. Subsequent measurements in the study group registered in the time window for DCI/vasospasm [1, 2]. showed a statistically significantly higher concentration of free radicals in patients who developed symptoms of DCI/vasospasm when compared to aSAH patients who did not present any of these complications.

Presented results may suggest the relevant contribution of free radicals in development of DCI and cerebral vasospasm. It has to be mark the currently published results are preliminary and the study is in progress.
